# Changes in canine serum N-glycosylation as a result of infection with the heartworm parasite *Dirofilaria immitis*

**DOI:** 10.1038/s41598-018-35038-7

**Published:** 2018-11-09

**Authors:** Anna-Janina Behrens, Rebecca M. Duke, Laudine M. C. Petralia, Sylvain Lehoux, Clotilde K. S. Carlow, Christopher H. Taron, Jeremy M. Foster

**Affiliations:** 1 New England Biolabs Inc., Ipswich, Massachusetts, 01938 USA; 2 Department of Surgery, Beth Israel Deaconess Medical Center, Harvard Medical School, Boston, MA 02115 USA

## Abstract

Filariases are diseases caused by infection with filarial nematodes and transmitted by insect vectors. The filarial roundworm *Dirofilaria immitis* causes heartworm disease in dogs and other carnivores. *D. immitis* is closely related to *Onchocerca volvulus*, *Wuchereria bancrofti* and *Brugia malayi*, which cause onchocerciasis (river blindness) and lymphatic filariasis (elephantiasis) in humans and are neglected tropical diseases. Serum N-glycosylation is very sensitive to both pathological infections and changes in mammalian biology due to normal aging or lifestyle choices. Here, we report significant changes in the serum N-glycosylation profiles of dogs infected with *D. immitis*. Our data derive from analysis of serum from dogs with established patent infections and from a longitudinal infection study. Overall, galactosylation and core fucosylation increase, while sialylation decreases in infected dog sera. We also identify individual glycan structures that change significantly in their relative abundance during infection. Notably, the abundance of the most dominant N-glycan in canine serum (biantennary, disialylated A2G2S2) decreases by over 10 percentage points during the first 6 months of infection in each dog analyzed. This is the first longitudinal study linking changes in mammalian serum N-glycome to progression of a parasitic infection.

## Introduction

*Dirofilaria immitis* is a filarial roundworm of considerable veterinary importance as the causative agent of heartworm disease in domestic dogs (*Canis lupus familiaris*) and cats (*Felis silvestris catus*). The parasite affects the pulmonary arterial system, the lung and the heart of canines and is often fatal^1–4^. Adult worms are capable of surviving in the arterial system of dogs for many years during which time they continually release microfilariae (*i.e*., L1 larvae) that circulate in peripheral blood vessels. Disease transmission occurs via mosquitos that feed on microfilaremic blood. Within the mosquito vector, microfilariae mature to infective L3 larvae that subsequently can be passed on to the next canine host while the mosquito takes its blood meal^4,5^. In the dogs, these larvae mature and patent infections (circulating microfilariae) are usually developed by 6–9 months after infection^4^.

Dogs can be asymptomatic for many years before signs of heartworm disease develop. Infections can be diagnosed by blood tests detecting either microfilariae or adult antigens as well as molecular methods. In clinical practice nowadays, a combination of antigen and microfilaria testing is recommended for diagnosis^6^. Detection of circulating antigens is mainly specific for the fecund female adult worm, which makes infection detectable only about half a year post-infection^7,8^. However, it is theoretically also possible to detect antibodies raised to parasite antigens present in earlier (pre-patent) phases of infection^9,10^.

Glycoproteins, *i.e*., proteins that are post-translationally modified by the attachment of O- or N-linked glycans, play important roles in many different biological functions^11^. Glycans are created by complex, non-template-driven biosynthetic pathways. As such, glycan structure can be modulated due to changes in gene expression patterns that occur in response to environmental changes. These changes in glycosylation range from fine-tuning biological processes to crucial contributions that enable novel biological functions^12^. Small changes in an N-glycan’s structure (such as the presence or absence of a core fucose) can have major impacts on the biological role of the protein. For example, the absence of core fucose on Fc N-glycans on immunoglobulin G (IgG) significantly alters its ability to elicit effector functions^13,14^. Serum glycosylation, while strikingly stable within individuals and highly regulated by hemostasis^15^, can change considerably with lifestyle, age and pathological conditions^16–18^. As such, serum glycan changes have been observed in association with cancer, inflammation, immunological diseases and congenital diseases^19–26^. Hence, there is a great diagnostic potential for glycobiomarkers, and the N-glycosylation analysis of easily sourced blood serum can be a valuable tool in the diagnosis of numerous diseases^25–32^ including infectious diseases which hitherto have been poorly studied from this perspective.

Here, we show the impact of parasitic infection with *D. immitis* on the total canine serum N-glycosylation profile. We observe remarkable changes in the abundances of core fucosylated, galactosylated and sialylated serum N-glycans when comparing healthy dogs to those that have established patent *D. immitis* infections. We further present the detailed analysis of a longitudinal cohort of infected dogs and are thus able to identify temporal glycan structural changes during infection. We propose that individual glycan peaks in canine serum N-glycan profiles as well as more global markers, such as the relative abundance of sialylated glycans, could potentially be considered as glycobiomarker candidates for *D. immitis* infection in dogs. We believe that our findings are relevant for the veterinary field as they suggest potentially new ways of understanding and diagnosing heartworm disease. Further, it is noteworthy that *D. immitis* is closely related to *O. volvulus*, *W. bancrofti* and *B. malayi*, causative agents of onchocerciasis and lymphatic filariasis afflicting more than 135 million humans^33,34^, thus making our results relevant for a wider filariasis research community. While alterations in IgG N-glycosylation have previously been associated with leishmaniasis^35^, asymptomatic filariasis^36^ and other parasitic infection in developing countries^37^ to the best of our knowledge, this is the first report of drastic changes in the mammalian total serum N-glycosylation profile as a consequence of parasitic infections.

## Results

### Characterization of serum N-glycans from healthy and diseased dogs

We have previously reported the detailed glycosylation analysis of canine serum^38^. To investigate the impact of filarial infection by *D. immitis* on the total N-glycosylation profile of serum, we analyzed serum samples from two different dog cohorts. In our first study, we analyzed sera from five dogs that had been infected with *D. immitis* for more than two years and thus carried a patent (i.e. active production of microfilariae) infection and compared them to sera from five healthy (control) dogs (Supplementary Table S1). In the second study, we investigated changes in serum N-glycosylation at initial stages of a *D. immitis* infection by analyzing a longitudinal serum collection sampled from four dogs collected over the first 27 weeks of infection (Supplementary Table S2)^39^. We performed serum N-glycosylation profiling of PNGase F-released and procainamide-labeled N-glycans by hydrophilic interaction chromatography-ultraperformance liquid chromatography (HILIC-UPLC) and further analyzed both serum from infected and uninfected dogs from the patent set by Matrix-assisted laser desorption/ionization time-of-flight mass spectrometry (MALDI-TOF MS).

While the patent set was collected in 2015, the longitudinal samples were collected in 1989 and stored at −80 °C since. Despite the 25-year age difference between the samples, the N-glycosylation profiles of the healthy/pre-infection serum samples from both cohorts were nearly identical (see Supplementary Fig. S1). There were only minor differences in the identified N-glycan peaks in the HILIC-UPLC spectra between the two sets (i.e. 42 glycan peaks were identified in total in the longitudinal set, compared to 44 in the patent set), which led to small differences in the peak numbering (for details see Supplementary Fig. S2 and Table S3).

### Alteration of serum N-glycosylation in dogs infected with *D. immitis*

**Figure 1:**
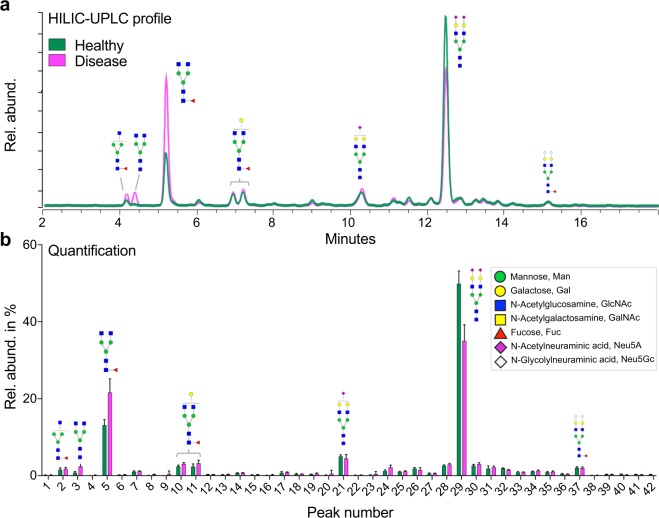
Serum N-glycosylation profile of dogs with a patent *D. immitis* infection compared to that of healthy dogs. (**a**) Representative HILIC-UPLC profile of enzymatically released and fluorescently labeled serum N-glycans from dogs carrying a patent *D. immitis* infection (pink) and of a healthy control sample (green). (**b**) Quantification of individual peaks derived from HILIC-UPLC spectra. Shown is mean + s.d of 5 biological replicates. Same color code as in panel a. See Supplementary Table S3 for peak identifications. Glycan structures of main peaks are annotated following the nomenclature outlined by the Consortium for Functional Glycomics (CFG). The inset in b shows the monosaccharide symbols used within this manuscript.

**Figure 2:**
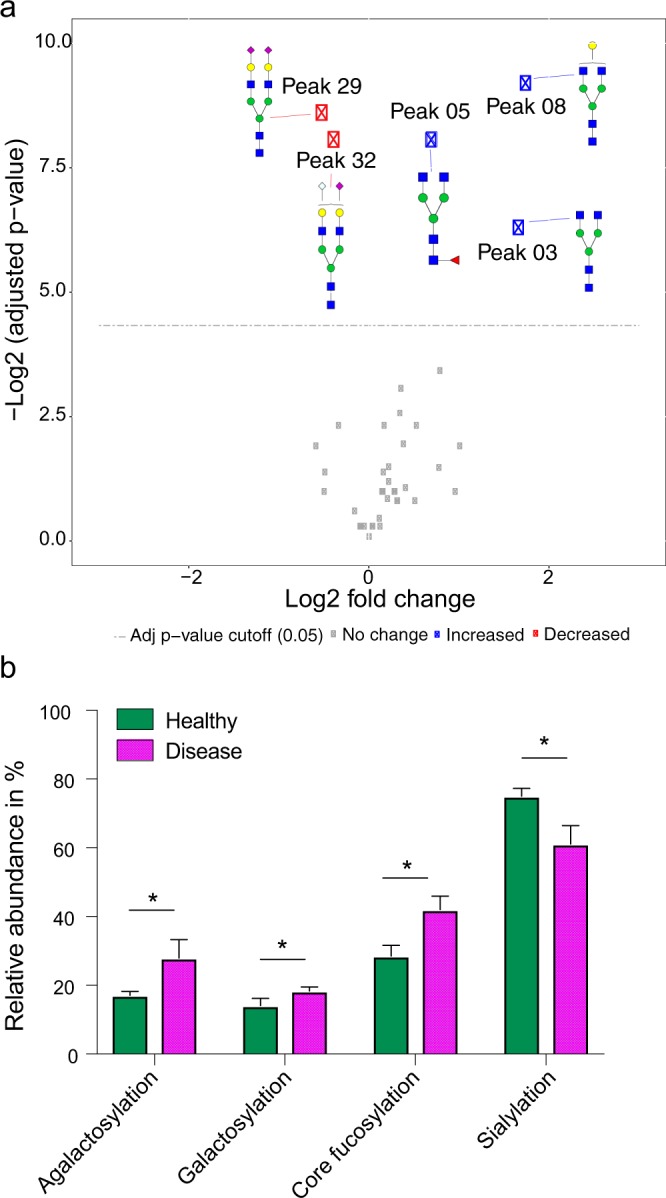
Significant changes of serum N-glycosylation in dogs with a patent *D. immitis* infection. Glycan peaks/classes were tested for significance using linear mixed-effects models. p-values were adjusted based on Benjamini and Hochberg method. The analysis is based on 5 biological and 2 technical replicates per group (healthy and disease; patent set). (**a**) Volcano Plot comparing serum N-glycan peaks from dogs infected with *D. immitis* to a healthy control group. Illustrated is the log2 fold change in glycan abundance and the negative log2 of adjusted p-values. The horizontal dashed line represents the adjusted p-value cutoff (0.05). The points above the dashed line are glycan peaks that decrease (red) and increase (blue) significantly. See Supplementary Table S4 for quantification data. (**b**) Significant changes in all analyzed glycan classes in dogs with a patent *D. immitis* infection. Glycan classes were identified and quantified by exoglycosidase digestion with α1-2,4,6 Fucosidase, β1-4 Galactosidase or α2-3,6,8 Neuraminidase. See Supplementary Fig. S4 for visualization of the glycan classes. Bar graphs show mean + s.d. Agalactosylation, adj. p-value = 0.001; galactosylation, adj. p-value = 0.003; core fucosylation, adj. p-value = 0.001; sialylation, adj. p-value = 0.001.

The N-glycosylation profiles of dogs that carry a patent *D. immitis* infection differ substantially from healthy dogs (Fig. 1). The most obvious differences are in the relative abundances of the two dominant glycan structures, the fucosylated biantennary FA2 (Peak No. 5) and the disialylated biantennary A2G2S2 (Peak No. 29; for a description of the glycan nomenclature, see legend to Supplementary Table S3). While the abundance of FA2 is considerably increased in diseased dogs, A2G2S2 is decreased. There were also obvious differences in less prominent structures (Fig. 1b). To investigate which of the N-glycan peaks significantly change in abundance within the patent set (consisting of 5 healthy and 5 infected dogs), we used linear mixed-effects models for statistical analysis. The volcano plot in Fig. 2a highlights the five peaks in the patent set that change significantly (adjusted p-value < 0.05). Among them are the two major peaks FA2 and A2G2S2, but also less abundant structures like A2 and A2G1. MALDI-TOF MS analysis of released and permethylated N-glycans from healthy and infected dogs confirms the changes in N-glycosylation as determined by HILIC-UPLC (Supplementary Fig. S3).

We were further interested in examining whether there are significant changes in serum glycosylation with regard to broader glycan classes. More precisely, we analyzed the significance of the change in abundance of core fucosylated, galactosylated (glycans containing at least one terminal galactose), agalactosylated (glycans without any galactoses) and sialylated glycans based on their sensitivity to α1-2,4,6 Fucosidase, β1-4 Galactosidase and α2-3,6,8 Neuraminidase, respectively. As can be seen in Fig. 2b, agalactosylation, galactosylation and core fucosylation are all significantly increased in dogs infected with *D. immitis*, whereas sialylation is decreased. Supplementary Fig. S4 illustrates the chromatographic peaks that were included in the quantification of the four glycans classes.

### Changes in canine serum N-glycosylation in a longitudinal *D. immitis* infection set

**Figure 3:**
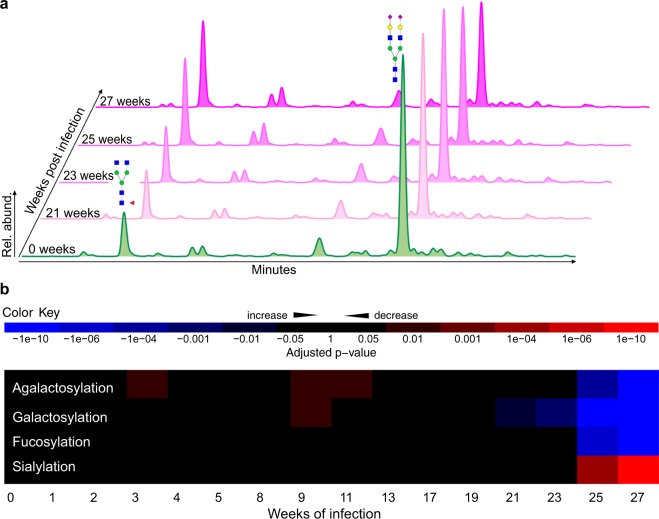
Longitudinal serum N-glycosylation profiles of *D. immitis* infection in dogs. (**a**) HILIC-UPLC profiles of enzymatically released and fluorescently labeled serum N-glycans from dog ID 116 (longitudinal set) from weeks 0, 21, 23, 25 and 27 post-infection with *D. immitis*. Rel. abund., relative abundance. The glycan structures of the two dominant peaks are annotated. (**b**) Heatmap of the changes in the abundance of serum N-glycan classes in *D. immitis* infection. Adjusted p-values were determined using linear mixed-effects models. Blue, increase; red, decrease. Glycan classes were identified and quantified by exoglycosidase digestion with α1-2,4,6 Fucosidase, β1-4 Galactosidase or α2-3,6,8 Neuraminidase. (Supplementary Fig. S3; Table S4).

**Figure 4:**
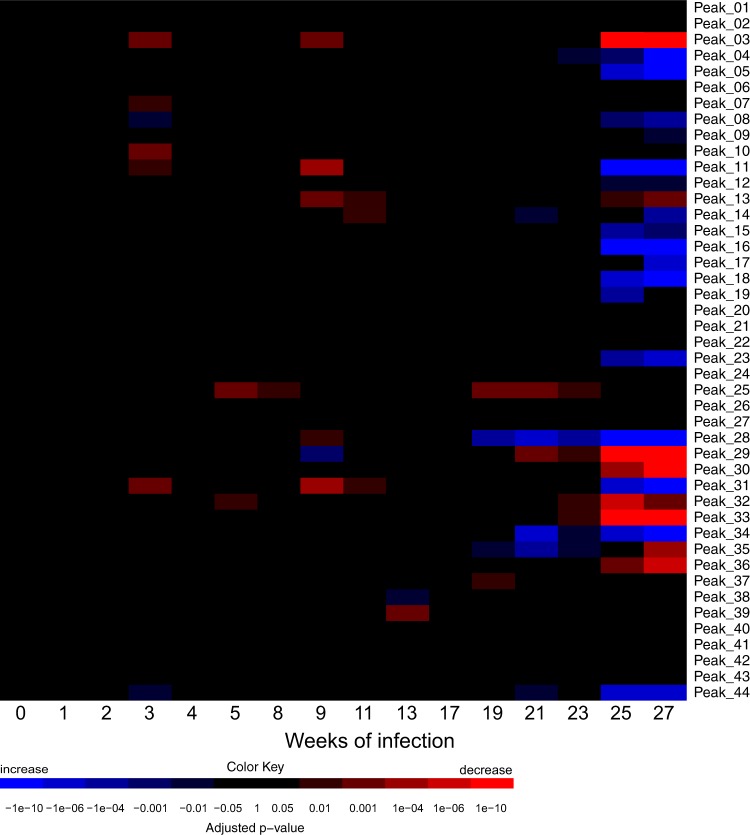
Heatmap of longitudinal canine serum N-glycosylation in *D. immitis* infection. Serum samples from four dogs were collected over 27 weeks post-infection. Significance was determined using linear mixed-effect models. Blue, increase; red, decrease. See Supplementary Table S3 for peak identifications. See Supplementary Table S4 for quantification data.

To investigate when the observed changes in canine serum N-glycosylation arise during the course of a parasitic infection with *D. immitis*, we analyzed a longitudinal serum set from four dogs over the first 27 weeks of infection (longitudinal set). The most noticeable changes arise after about 25 weeks post-infection. The relative abundance of the main peak (A2G2S2) significantly drops, whereas we see an increase in FA2 (Fig. 3a). In fact, 22 out of 44 chromatographic N-glycan peaks in this longitudinal set change significantly (adjusted p-value < 0.05) 25 weeks post *D. immitis* infection (Fig. 4). Similar to what we observed in the patent set, all four glycan classes also show significant changes after 25 weeks of infection (Fig. 3b).

Interestingly, the serum N-glycosylation profiles transiently change after about 3 weeks and 9 weeks of infection, where we see a general decrease in mainly small, agalactosylated glycans as well as galactosylated structures (Figs 3b and 4). This is somewhat inverted at later timepoints, where, broadly speaking, we see a general increase in smaller glycans (*i.e*., agalactosylation and galactosylation) and also in fucosylation. Peak 28, which contains the biantennary, monosialylated (Neu5Gc) glycan FA2G2S_Neu5Gc_1, is consistently and significantly decreased in comparison to healthy dog serum after only 19 weeks of infection (Fig. 4), thus making it a potential biomarker candidate for early infection. However, we could not identify a similar clear change in the relative abundance of this particular glycan in dogs with a patent *D. immitis* infection.

### The N-glycosylation profile of serum IgG is unaltered

We purified IgG from healthy and infected dogs (patent set) using Protein G and performed N-glycosylation profiling by HILIC-UPLC. The IgG N-glycan profiles of healthy and infected dogs look almost identical (Supplementary Fig. S5) and we did not determine any statistical changes in the abundances of canine IgG glycan structures. However, we further determined the concentration of IgG in the serum samples of the patent set using ELISA, and found the average IgG concentration to be about 1.5 times higher in dogs with a patent infection compared to the healthy controls. Thus, a change in the relative abundance of serum glycoproteins likely contributes to the observed changes in canine N-glycosylation upon infection with *D. immitis*.

## Discussion

We observed remarkable changes in the total serum N-glycosylation profiles of dogs infected with *D. immitis*. The relative abundance of sialylated N-glycans decreases whereas the level of galactosylation and fucosylation increased. The most obvious and dominant changes occurred as early as 25 weeks post-infection, although we observe some fluctuation in serum N-glycosylation as early as 3 weeks post-infection. Generally, changes in serum N-glycosylation can be attributed to either a change in the glycan microheterogeneity of one or more glycoproteins or to a change in the total abundance of glycoproteins in serum. Among the N-glycan structures that change the most in dogs infected with *D. immitis* are FA2 (increase) and A2G2S2 (decrease). FA2 has been shown to predominantly originate from IgG^40,41^. While a previous study has reported changes in the IgG sialylation in people infected with a related filarial nematode, *W. bancrofti*^36^, our analysis revealed no change in IgG N-glycan microheterogeneity in dogs with a patent *D. immitis* infection. We do, however, see an increased abundance of serum IgG in infected dogs that is likely responsible for at least part of the change in the relative abundances of serum N-glycans. We hypothesize that *D. immitis* may use a long-term polyclonal IgG response as an obfuscation tactic. Along these lines, increased levels of serum IgG have been previously reported in patients infected with *Leishmania donovani*^42^, where changes in IgG N-glycosylation have been associated with disease severity^35^. Helminths as such have been shown to modulate and suppress immune responses by regulating certain types of T cells^43^. It is highly likely that the observed effects are caused by multiple serum glycoproteins, but this requires further investigation.

Changes in the abundance of FA2 in blood serum have previously been associated with conditions like liver fibrosis^28^, pregnancy in patients with rheumatoid arthritis^44^ and peritoneal dialysis patients^31^. Possibly the most pronounced increases of FA2 in human serum have been reported to correlate with aging in both males and females^17,45^. The authors hypothesized that the observed change could possibly be explained by increased activity of β-galactosidase activity as they also observed a decrease in FA2G2. We, however, did not observe any decrease in FA2G or FA2G2. On the contrary, corresponding peaks were elevated in the longitudinal set after 25 weeks (Peak No. 12 and Peak No. 16) and showed no significant change in the patent set. Thus, it is highly unlikely that the changes observed in our study are age-related or caused by similar effects in enzyme activity.

In this study, we analyzed two separate sets of canine serum infected with *D. immitis*; a cohort with a patent infection and a longitudinal study over the first 27 weeks of infection. We observed a larger number of glycan structures that showed significant changes after 25 weeks of infection in the longitudinal set (22 peaks) compared to five peaks in the patent set. Given the time span of about 25 years between the age of the two sources of sera and the different study designs, particularly the maturity of the heartworm infections, results are only comparable to some degree. Encouragingly, the observed changes in serum N-glycan peak relative abundances upon infection with *D. immitis* are broadly the same in both studies (*i.e*., decrease in sialylation, increase in galactosylation and fucosylation and the larger relative abundance of FA2). Further, all five glycan peaks that showed significant changes in the patent set after 2.5 years of infection were also significantly altered towards the end of the longitudinal study (25 weeks post-infection) and thus present potential biomarker candidates. It appears that serum N-glycosylation is more affected during the initial phase of *D. immitis* infection, likely reflecting immune responses as the larvae mature to adulthood, migrate into the heart and the pulmonary arterial system and start producing microfilariae. More precisely, it has been reported that after about 3 weeks of infection, when we see the first changes in serum N-glycosylation (Fig. 4), most larvae have migrated from the subcutaneous tissues to the abdomen^4,5^. They reach the heart as early as week 10 of infection, which is roughly when we see further distinct changes in the heatmap in Fig. 4. Dogs usually develop patent infections where they actively produce microfilariae 6–9 months post-infection^4,5^, which coincides with the time point where we observe the beginning of the most pronounced changes in the serum N-glycosylation profiles (25 weeks; Fig. 4). While some of the changes in serum N-glycosylation might be transient reflections of canine immune responses to *D. immitis* infection, it appears that the quantitatively most significant changes remain present over the longer course of infection as it is in the case of the patent set.

Given that blood is an easily sampled body fluid and that we observe changes in broad glycan classes one could imagine potential glycobiomarker candidates for diagnosing or monitoring *D. immitis* infection. Detection assays could possibly be based on lectin-based quantification capable of detecting terminal glycan epitopes such as e.g. sialylation or fucosylation. Of course, it needs to be further investigated which (if any) of these serum N-glycan changes are specific to a canine *D. immitis* infection and if infected dogs in a more natural infection setting show equally pronounced glycosylation changes. The dog breed, the presence or absence of microfilariae and the severity of the disease could very well influence the intensity of glycosylation changes. It also needs to be considered that the abundance of serum FA2 in dogs could be similarly affected by aging as it is the case in humans^17,45^.

To the best of our knowledge, this is the first report of significant and drastic serum N-glycosylation changes during a nematode infection. In the light of the fact that *D. immitis* is closely related to the human parasites *O. volvulus*, *W. bancrofti* and *B. malayi*, future studies should investigate if similar drastic changes in serum N-glycosylation also occur in human nematode infections.

## Methods

### Canine serum samples

Canine serum samples originated from beagle dogs and were purchased from TRS Laboratories Inc (Athens, GA, USA). All animal experiments were approved by the IACUC committee of TRS Laboratories Inc. and in compliance with the USDA Animal Welfare Act. This study comprises the analysis of sera originating from two independent canine cohorts that were infected with *D. immitis*. The first cohort, termed *patent set*, contains five healthy (control) dogs and 5 diseased dogs. The 5 diseased dogs were infected via transplantation of adult worms of one of the *D. immitis* strains ‘Wildcat’, ‘Pepper’ or ‘Georgia II’ into the jugular vein. Serum samples were drawn after ~2.5 years of infection. More detailed information on this set can be found in Supplementary Table S1. The second cohort of serum samples, termed *longitudinal set*, originated from a previously published longitudinal study of dogs infected with *D. immitis*^39^. In brief, 4 beagle dogs were subcutaneously infected with L3 larvae at week 0 and week 7. Blood samples were collected for a period of 27 weeks of infection. More detailed information on this set can be found in Supplementary Table S2. Serum samples were stored at −80 °C.

### Purification of IgG from canine serum

IgG was purified from canine serum using Protein G magnetic beads according to the manufacturers’ instructions (New England Biolabs).

### Determination of IgG concentration

The concentration of IgG in canine serum was determined by an ELISA assay Kit (Canine IgG ELISA Kit, Abcam) according to the manufacturer’s instructions.

### N-glycan analysis by HILIC-UPLC

N-glycans were released from serum or IgG using Rapid PNGase F and fluorescently labeled with procainamide as described previously^38^. The procainamide labeled N-glycans were analyzed by HILIC-UPLC with fluorescence and mass detection on a Waters Acquity H-class instrument composed of a binary solvent manager, a sample manager, a fluorescence detector (excitation wavelength 310 nm; detection wavelength 370 nm) and a QDa mass detector (settings: positive mode; target sampling rate: 10 point/sec; gain: 1; capillary voltage: 1.5 kV; probe temperature: 600 °C). Glycans were separated using an Acquity BEH Amide Column (130 Å, 1.7 μm, 2.1 mm × 150 mm; Waters) with 50 mM ammonium formate, pH 4.4 as solvent A and acetonitrile as solvent B using a linear gradient of 70% to 53% solvent B at 0.56 ml/min for 25 min. Data acquisition, processing and analysis was performed using Empower 3 software (Waters).

### Exoglycosidase digestion of released glycans

Digestion of released glycans with a panel of recombinant exoglycosidases was performed to quantify the abundance of individual glycan classes (e.g. those carrying sialic acids) as well as to confirm glycan structures via sequential digestion as described previously^38^. In brief, glycans were digested with α2-3,6,8 Neuraminidase, α1-2,4,6 Fucosidase O, β1-4 Galactosidase, β*-N*-Acetyl-Glucosaminidase and α1-2,3,6 Mannnosidase (all from New England Biolabs) according to the manufacturer’s instructions. Samples were then analyzed by HILIC-UPLC (see above).

### Glycan release, permethylation and MALDI-TOF MS analysis

N-glycans were released with PNGase F, subsequently permethylated and analyzed by MALDI-TOF MS in positive ion mode on an UltraFlex II MALDI-TOF mass spectrometer (Bruker) as described in detail previously^38^. Glycan structures were assigned manually, based on known biosynthesis pathways and with the help of GlycoWorkbench^46^.

### Data analysis

Statistical data analysis was performed using R 3.5.0 in an environment of R studio 1.1.447. The impact of *D. immitis* infection on canine serum glycosylation was explored using linear-mixed effects models. Peak areas of HILIC-UPLC glycan spectra were quantified using Empower 3 (Waters). The peak areas within one spectrum were normalized to the same arbitrary total peak area of 100000 among all spectra. The R package MSstats^47^ was utilized for data processing (including log transformation) and statistical modeling. MSstats employs the lm and lmer functionalities in R, but customizes the statistical design i.e. group comparison (patent set) or time course experiments (longitudinal set) based on the input data.

## Electronic supplementary material

Supplementary Information

Supplementary Table S4

## Data Availability

All data generated and analyzed during this study are included in this published article and its Supplementary Information files.
