# Supplementary material for: Changes in canine serum N-glycosylation as a result of infection with the heartworm parasite Dirofilaria immitis
Source: Sci Rep. 2018 Nov 9;8:16625. doi: 10.1038/s41598-018-35038-7 (PMC6226445; doi:10.1038/s41598-018-35038-7)
Supplement: Supplementary file 1 — Supplementary Information [file 41598_2018_35038_MOESM1_ESM.pdf]

Supplementary Information for

Changes in canine serum N-glycosylation as a result of infection with the heartworm parasite *Dirofilaria immitis*

Anna-Janina Behrens[1*], Rebecca M. Duke[1], Laudine M. C. Petralia[1], Sylvain Lehoux[2], Clotilde K. Carlow[1], Christopher H. Taron[1], Jeremy M. Foster[1*]

[1]New England Biolabs Inc., Ipswich, Massachusetts 01938, USA

[2]Department of Surgery, Beth Israel Deaconess Medical Center, Harvard Medical School, Boston, MA, 02115, USA

*To whom correspondence should be addressed, Anna-Janina Behrens, annaj.behrens@gmail.com; Jeremy M. Foster, foster@neb.com;

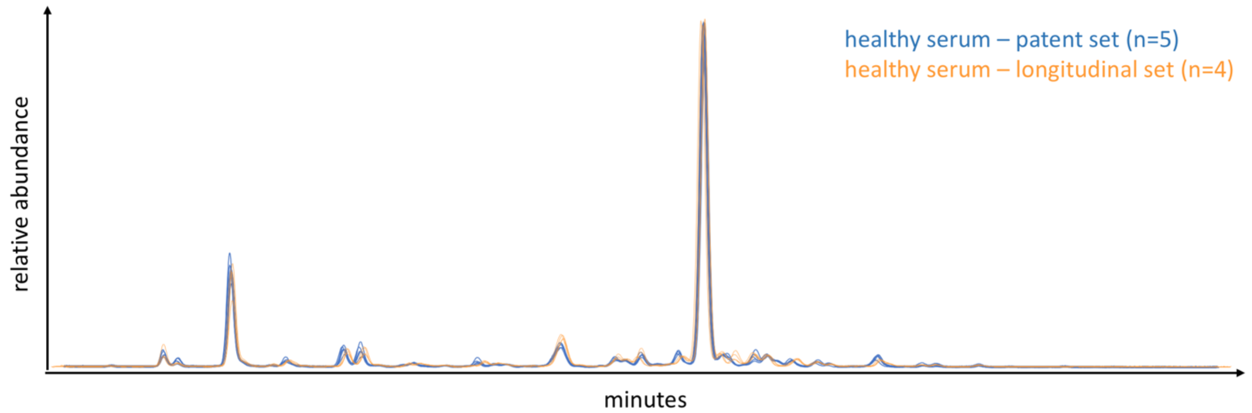

Figure S1. Comparison of serum N-glycosylation profiles of healthy dogs from both study cohorts. Longitudinal study (orange) was conducted in 1989. The patent set (blue) was conducted in 2015. Sera were stored at -80 °C and analyzed at different time points in 2017 on the same UPLC instrument.

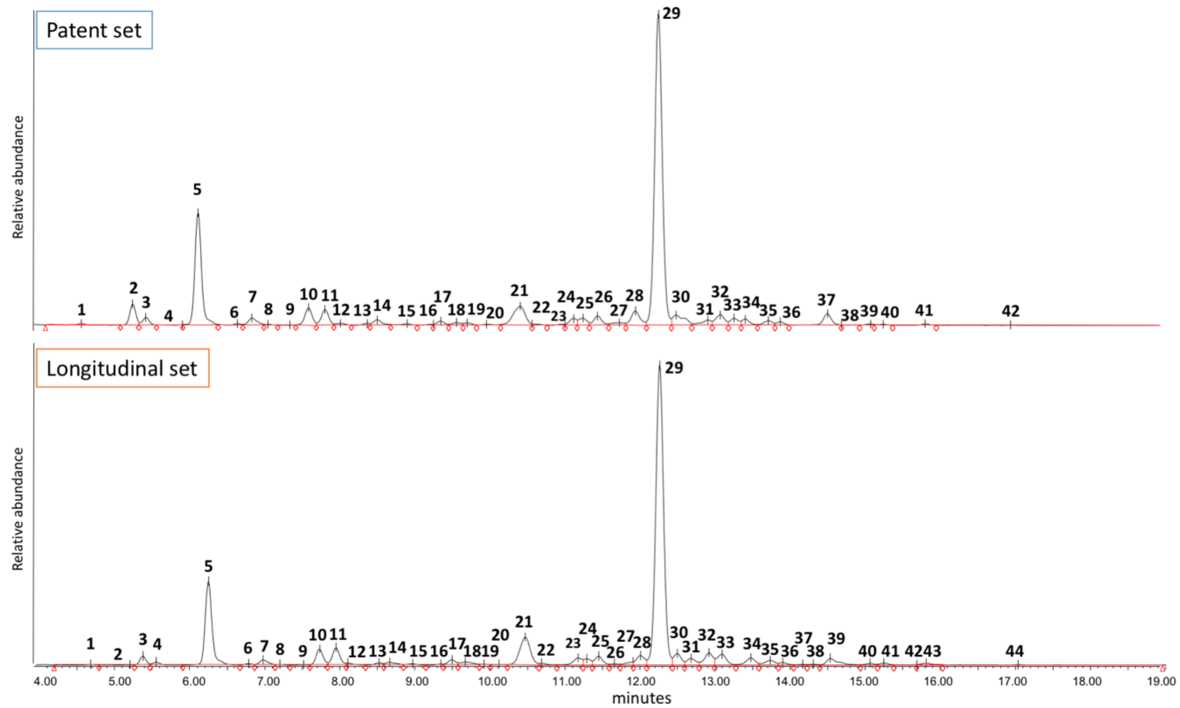

Figure S2. Peak numbering of patent and longitudinal set. See Supplementary Table S3 and Behrens *et al.*, 2018.

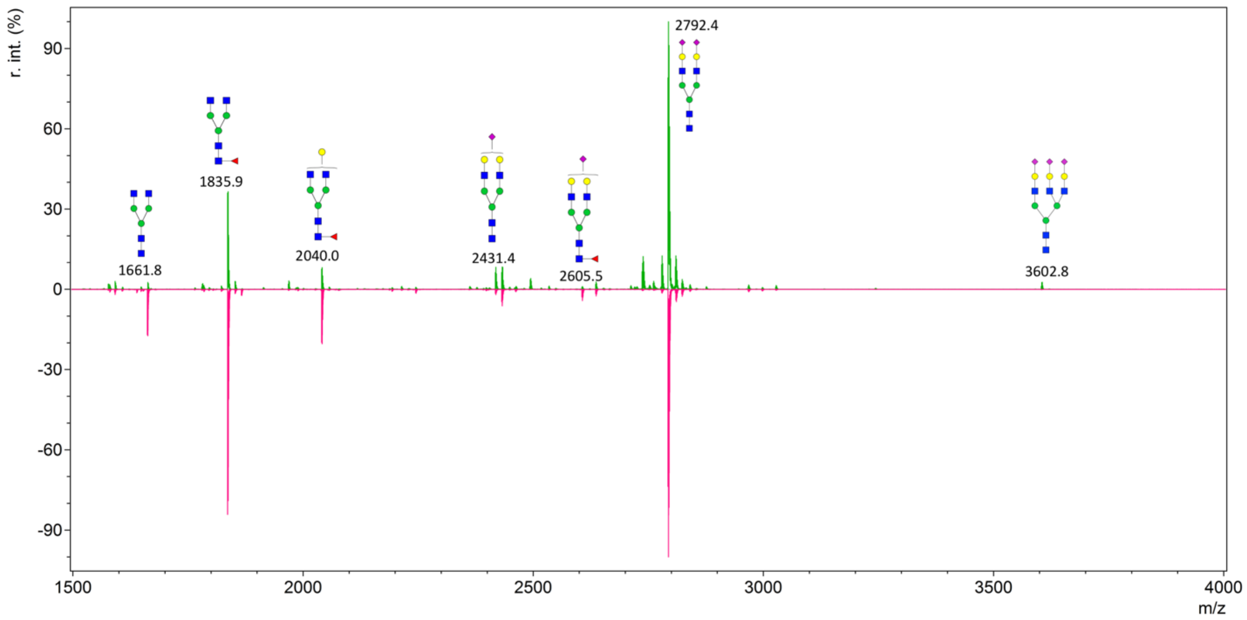

Figure S3. MALDI-TOF MS spectra of changes in serum N-glycans in a patent *D. immitis* infection. Healthy dog serum (green; dog ID 172) infected dog serum (pink; flipped spectrum; dog ID 645). Main peaks are annotated. For a detailed information on peak assignments, see Behrens *et al*., 2018[1].

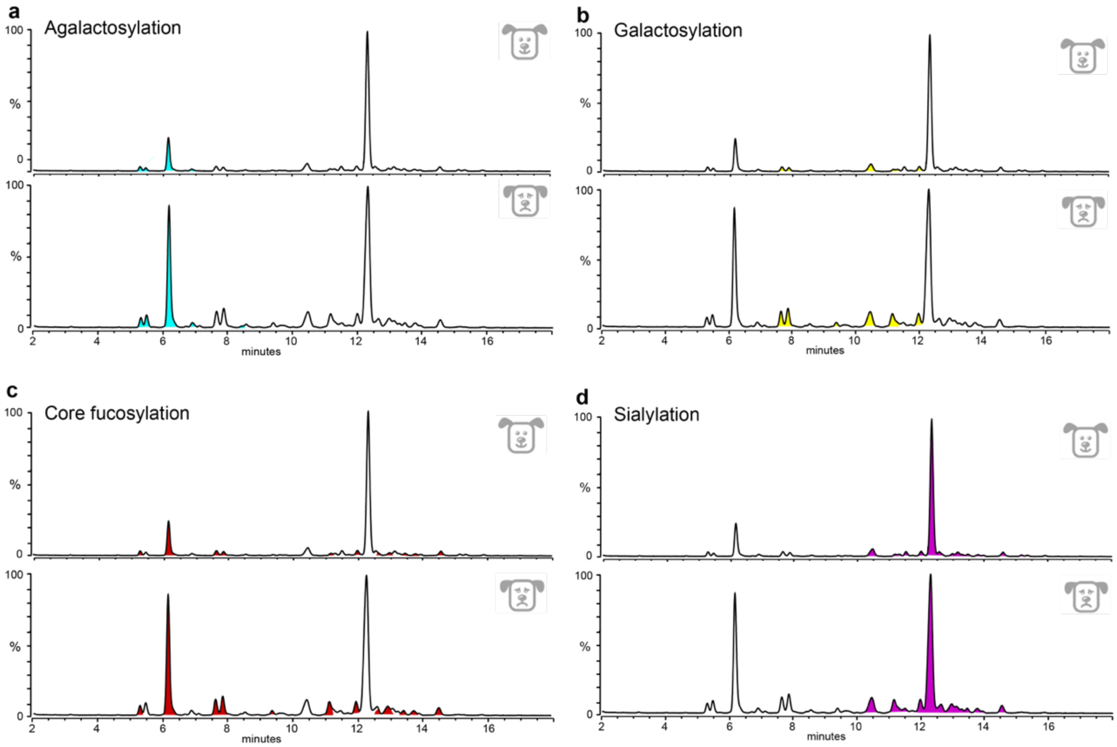

Figure S4. Visualization of glycan classes in canine serum HILIC-UPLC spectra. Glycan classes were identified and quantified by exoglycosidase digestion with α1-2,4,6 Fucosidase, β1-4 Galactosidase or α2-3,6,8 Neuraminidase. The spectra visualization in this figure is adjusted to the height of the largest peak, thus, total subjective peak areas between healthy (top panels) and diseased (bottom panels) dogs may vary.

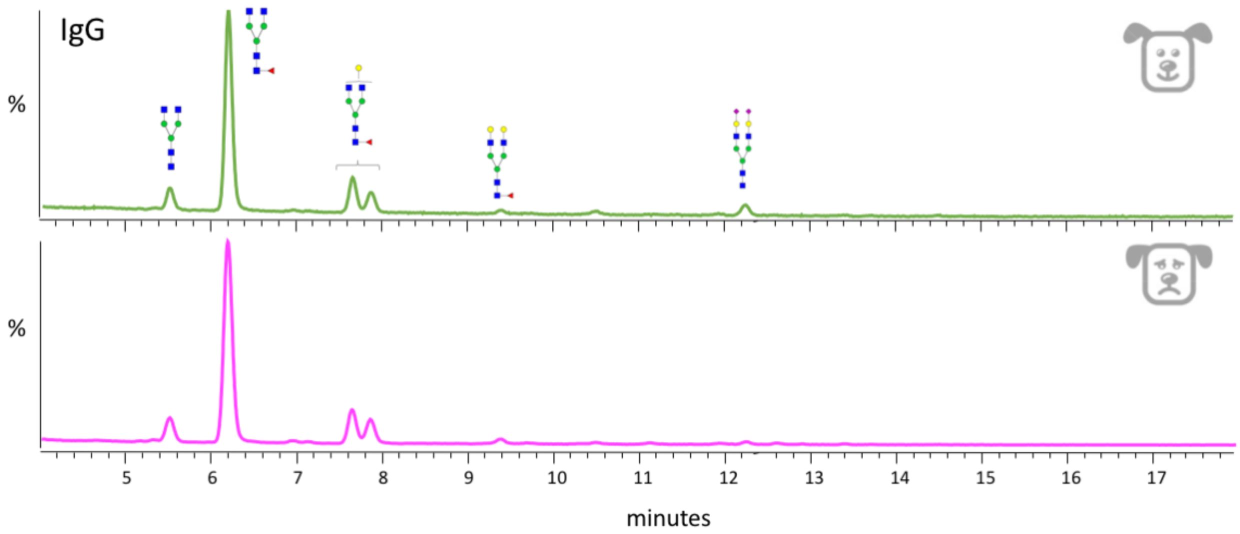

Figure S5. Representative N-glycosylation profiles of IgG purified from healthy and diseased dog serum. IgG was purified from canine serum using Protein G. Upper panel, IgG purified from dog ID 172 (control; patent set); lower panel, IgG purified from dog ID 645 (disease; patent set). The detailed glycan structures in canine IgG have been previously reported[1].

Table S1. Overview of patent set of infected dogs. ID refers to the identification number as assigned by TRS Laboratories Inc. Dogs 010, 172, 211, 424 and 487 are uninfected controls. y, years; m, months; Mf, microfilaria. Adult worms were transplanted in pairs (as listed in # worms) via the jugular vein. Age of these worms at time of transplantation is shown. The microfilaria counts refer to the date the blood was drawn. Study was conducted 2015/2016.

ID	*Gender*	*Strain*	*# worms*	*Age (worms)*	*Duration of infection*	*Mf/20 µl*
010	male	uninfected				
172	female	uninfected				
211	male	uninfected				
424	male	uninfected				
487	male	uninfected				
571	male	Wildcat	15 pairs	7.5 months	2y 5m	805
583	female	Wildcat	15 pairs	7.5 months	2y 5m	892
634	male	Pepper	10 pairs	9.5 months	2y 2m	288
635	male	Georgia II	10 pairs	7 months	2y 4m	822
645	female	Georgia II	10 pairs	7 months	2y 4m	1090

Table S2. Overview of longitudinal set of infected dogs. ID refers to the identification number as assigned by TRS Laboratories Inc. This longitudinal study was performed in 1989. The beagles were subcutaneously infected with L3 larvae of the TRS strain at weeks 0 and 7. For more detailed information, refer to Mejia *et al*., 1994[2]. Mf, microfilaria. ml; milliliter. Parasites (adult worms) were recovered in the heart on week 27 at the end of the study. The microfilariae counts in the blood refer to the end of the longitudinal study, i.e., 27 weeks post-infection.

ID	***Gender***	***Mf/ml***	***Recovered adult worms***
116	female	12	32
117	female	131	36
120	male	0	30
121	male	0	41

Table S3. List of identified glycan structures in the HILIC-UPLC chromatograms of N-glycans. N-glycans were released from serum samples from the patent (a) and the longitudinal (b) set. See Supplementary Figure S2 for peak numbering. Glycan names are constructed as follows: Mn = number (n) of mannose residues; An = number (n) of antennae (e.g. A3 = triantennary); Gn = number (n) of galactose residues; F in the front of the name indicates the presence of a core fucose; S: number of Neu5Ac; S_{Gc}: number of Neu5Gc. Linkages are indicated in brackets if known. RT, retention time exp., experimental; calc., calculated; Hex, Hexose; HexNAc, *N*-acetylhexosamine; Fuc, Fucose; n.i., not identified. Glycan structures are annotated following the nomenclature outlined by the Consortium for Functional Glycomics (CFG). Monosaccharide codes as in inset Figure 1B. *m/z* values of procainamide-labeled glycans are listed when visible in the QDA mass detector.

**Peak ID**	**RT**	***m/z* exp.**[1]	***m/z* calc.**[1]	**Main identified glycan(s)**	**Proposed (main) structure**
**(a) patent set**					
**1**	4.6	586.4	586.3	A1	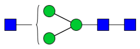
**2**	5.3	740.4	740.3	F(6)A1	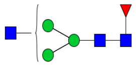
**3**	5.5	768.8	768.8	A2	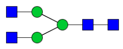
**4**	5.8	768.8	768.8	A2	
**5**	6.2	842.0	841.9	F(6)A2	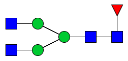
**6**	6.6	821.5	821.4	F(6)A1(6)G1	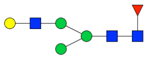

**7**	6.9	727.7/848.8	727.8/849.9	M5/A2(6)G1	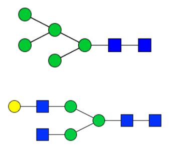
**8**	7.1	848.8	849.9	A2(6)G1	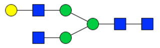
**9**	7.3	848.8	849.9	A2(3)G1	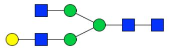
**10**	7.6	923.1	923.2	F(6)A2(6)G1	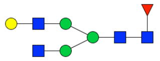
**11**	7.8	923.1	923.2	F(6)A2(3)G1	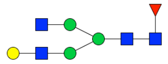
**12**	8	923.1	923.2	F(6)A2(3)G1	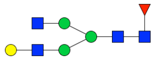
**13**	8.4	808.8/902.3	808.8/902.3	M6/minor: Hex5HexNAc3Fuc1 (Hybrid)	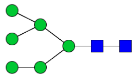
**14**	8.5	808.8/931.1	808.8/931.1	M6/minor: A2G2	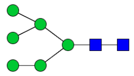
**15**	8.8	995.4	995.4	A2G1(6)S1	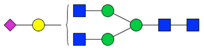
**16**	9.2	1003.9/1068.4	1003.9/1068.4	F(6)A2G2/F(6)A2G1S1	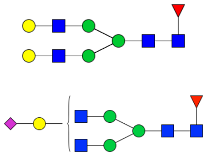
**17**	9.3	1003.9	1003.9	F(6)A2G2	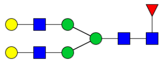
**18**	9.5	974.9/1068.4	974.9/1068.4	Hybrid (M4A1G1S1 or F(6)A1G1S_{gc}1)/minor: F(6)A2G1S1	
**19**	9.7	974.9/1063.9	974.9/1063.9	M4A1G1S1 or F(6)A1G1S_{gc}1 + Hybrid (M5A1G1S_{gc}1)	
**20**	9.9	889.8	889.9	M7	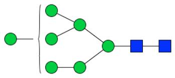
**21**	10.3	1076.5	1076.5	A2G2(6)S1	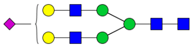
**22**	10.5	1076.5	1076.5	A2G2S1	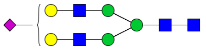
**23**	10.7			n.i.	
**24**	11.0	1149.6	1149.5	F(6)A2G2(6)S1	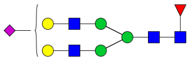

**25**	11.1	1084.4/1055.9	1084.4/1055.9	A2G2S$_{gc}$1/minor: F(6)M4A1G1S$_{gc}$1	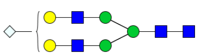
**26**	11.5	1222.0	1222.0	A2G2(3/6)S2	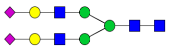
**27**	11.7	1064.5	1064.4	F(6)M6A1or F(6)M7A1	
**28**	12.0	1157.5	1157.5	F(6)A2G2S$_{gc}$1	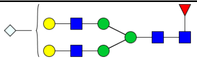
**29**	12.3	1222.0	1222.0	A2G2(6/6)S2	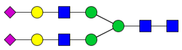
**30**	12.5	1222.0/1166.4	1222.0/1166.4	A2G2S2/F(6)A2G4	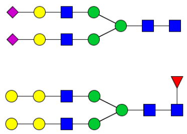
**31**	12.9	1295.0	1295.0	F(6)A2G2(6/6)S2	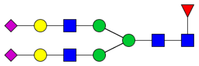
**32**	13.2	1230.1/1238.1	1230.0/1238.0	A2G2S1S$_{Gc}$1/minor: A2G2S$_{Gc}$2	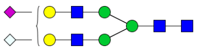
**33**	13.3	1238.1	1238.0	A2G2S$_{Gc}$2	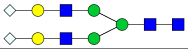
**34**	13.5	1238.0/1230.5	1238.0/1230.5	A2G2S$_{Gc}$2/F(6)A2G3S1	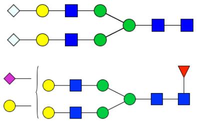
**35**	13.8			F(6)A2G2S1S$_{Gc}$1	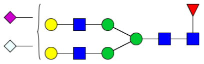
**36**	14.1			F(6)A2G2S$_{Gc}$2	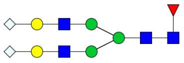
**37**	14.5	1113.7	1113.7	F(6)A2G2S$_{Gc}$2/A3G3	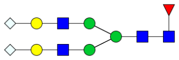
**38**	14.7			multiantennary structures, mainly A3G3 and A3G3S3	
**39**	14.8				
**40**	15.0				
**41**	15.9				
**42**	17.0				
**(b) longitudinal set**					
**1**	4.6	586.4	586.3	A1	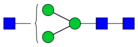
**2**	5.1	740.3	740.3	F(6)A1	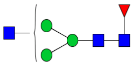
**3**	5.3	740.3	740.3	F(6)A1	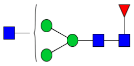
**4**	5.5	768.9	768.8	A2	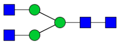
**5**	6.2	842.0	841.9	F(6)A2	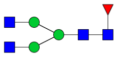

**6**	6.6	821.5	821.4	F(6)A1(6)G1	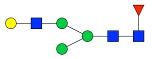
**7**	6.9	727.7/848.8	727.8/849.9	M5/A2(6)G1	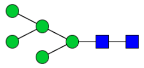 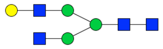
**8**	7.1	848.8	849.9	A2(6)G1	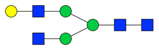
**9**	7.3	848.8	849.9	A2(3)G1	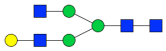
**10**	7.6	923.2	923.2	F(6)A2(6)G1	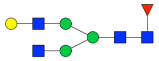
**11**	7.8	923.1	923.2	F(6)A2(3)G1	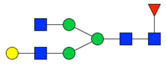
**12**	8	923.1	923.2	F(6)A2(3)G1	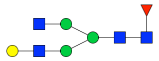
**13**	8.4	808.8/902.3	808.8/902.3	M6/minor: Hex5HexNAc3Fuc1 (Hybrid)	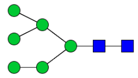
**14**	8.5	808.8/931.1	808.8/931.1	M6/minor: A2G2	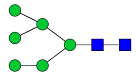
**15**	8.8	995.4	995.4	A2G1(6)S1	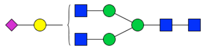
**16**	9.2	1003.9/1068.4	1003.9/1068.4	F(6)A2G2/F(6)A2G1S1	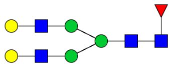 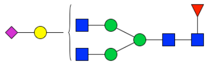
**17**	9.3	1003.9	1003.9	F(6)A2G2	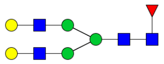
**18**	9.5	974.9/1068.4	974.9/1068.4	Hybrid (M4A1G1S1 or F(6)A1G1S_{gc}1)/minor: F(6)A2G1S1	
**19**	9.7	974.9/1063.9	974.9/1063.9	M4A1G1S1 or F(6)A1G1S_{gc}1 + Hybrid (M5A1G1S_{gc}1)	
**20**	9.9	889.8	889.9	M7	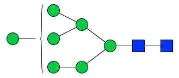
**21**	10.3	1076.5	1076.5	A2G2(6)S1	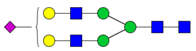
**22**	10.5	1076.5	1076.5	A2G2S1	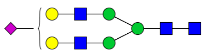

**23**	11.0	1149.6	1149.5	F(6)A2G2(6)S1	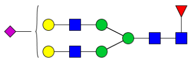
**24**	11.1	1084.4/1055.9	1084.4/1055.9	A2G2S_{gc}1/minor: F(6)M4A1G1S_{gc}1	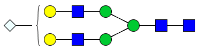
**25**	11.5	1222.0	1222.0	A2G2(3/6)S2	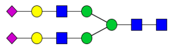
**26**	11.7	1064.5	1064.4	F(6)M6A1or F(6)M7A1	
**27**	11.8	970.9	970.9	M8	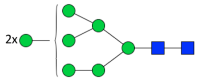
**28**	12.0	1157.5	1157.5	F(6)A2G2S_{gc}1	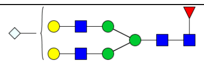
**29**	12.3	1222.0	1222.0	A2G2(6/6)S2	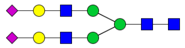
**30**	12.5	1222.0/1166.4	1222.0/1166.4	A2G2S2/F(6)A2G4	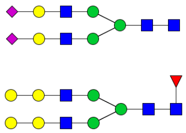
**31**	12.6	1295.0/1230.0	1295.0/1230.0	F(6)A2G2S2/A2G2S1S_{Gc}1	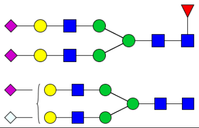
**32**	12.9	1295.0	1295.0	F(6)A2G2(6/6)S2	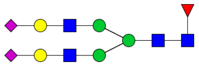
**33**	13.2	1230.1/1238.1	1230.0/1238.0	A2G2S1S_{Gc}1/minor: A2G2S_{Gc}2	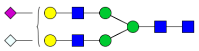
**34**	13.5	1238.0/1230.5	1238.0/1230.5	A2G2S_{Gc}2/F(6)A2G3S1	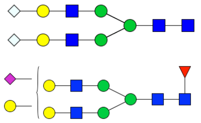
**35**	13.8			F(6)A2G2S1S_{Gc}1	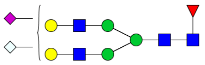
**36**	14.1			F(6)A2G2S_{Gc}2	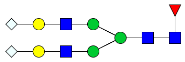
**37**	14.2			multiantennary, sialylated glycans	
**38**	14.4				
**39**	14.5	1113.7	1113.7	F(6)A2G2S_{Gc}2/A3G3	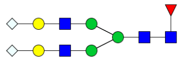
**40**	15.0			multiantennary structures, mainly A3G3 and A3G3S3	
**41**	15.2				
**42**	15.7				
**43**	16.2				
**44**	17.0				

[1]$(M+H)^{2+}$

Table S4. Quantification data – HILIC-UPLC. Listed are the relative abundances of peak areas in % for the patent (a) and longitudinal (b) dog serum sets. Peak areas were quantified using Empower 3 from HILIC-UPLC spectra. The relative abundances are in relation to the total peak area within one spectrum. The second sheet contains the glycan category quantification data. Table is in a separate Excel file.

1 Behrens, A.-J. *et al.* Glycosylation profiling of dog serum reveals differences compared to human serum. *Glycobiology*, (2018).

2 Mejia, J. S. & Carlow, C. K. An analysis of the humoral immune response of dogs following vaccination with irradiated infective larvae of *Dirofilaria immitis*. *Parasite Immunol.* **16,** 157-164, (1994).
